# Effects of Transcutaneous Vagus Nerve Stimulation, Neurofeedback, and their Combination on Cortisol, Anxiety, and Depression Subtypes in Non-Clinical Adults

**DOI:** 10.1007/s10484-025-09764-4

**Published:** 2026-01-06

**Authors:** Seyedeh Zeinab Molaeizadeh, Aitor Aritzeta Galan, Zahra Zolghadr

**Affiliations:** 1 Department of Psychology, University of the Basque Country (UPV/EHU), San Sebastian, Spain; 2 National Center for Health Insurance Research, Tehran, Iran

**Keywords:** Neurofeedback, Transcutaneous vagus nerve stimulation, Anxiety, depression, Alpha / theta ratio, Cortisol, Neuromodulation

## Abstract

Non-invasive neuromodulation techniques such as neurofeedback (NF) and transcutaneous vagus nerve stimulation (tVNS) are attracting growing interest for their potential to influence brain activity and emotional regulation. This pilot study investigated the individual and combined effects of these methods on psychological measures, electrophysiological markers, and salivary cortisol. Twenty healthy young adults (19 females, 1 male, aged 18–24) participated, with outcomes including psychological measures (anxiety and depression), EEG Alpha / Theta ratio, and salivary cortisol. NF was associated with significant reductions in anxiety and depressive symptoms. Notably, despite the NF protocol directly targeting Alpha / Theta oscillations, a significant increase in the Alpha / Theta ratio was observed only in the tVNS group, indicating a distinct bottom-up influence on neural dynamics. The combined intervention did not yield additional benefits, and cortisol levels remained unchanged. These preliminary findings suggest that NF and tVNS may operate through distinct pathways—NF influencing psychological outcomes via top-down regulation and tVNS modulating electrophysiological activity via bottom-up pathways. The absence of sham control conditions limits causal attribution of effects. Given the small sample size (*n* = 5 per group) and exploratory nature of this study, these results should be interpreted as hypothesis-generating and require replication in larger, adequately powered trials.

**Supplementary Information:**

The online version contains supplementary material available at https://doi.org/10.1007/s10484-025-09764-4.

## Introduction

Anxiety and depression are among the most common mental health conditions worldwide and contribute substantially to disability and reduced quality of life (World Health Organization, 2019). While pharmacological treatments are widely available, many patients experience limited efficacy, adverse effects, or poor adherence (Cui, 2015; Szuhany and Simon, 2022), motivating the search for safe and effective non-pharmacological approaches.

Neurofeedback (NF) and transcutaneous vagus nerve stimulation (tVNS) are two non-invasive neuromodulation methods that have been studied for their effects on emotional regulation and neural dynamics. NF provides individuals with real-time feedback on their brain activity, allowing self-regulation of neural oscillations. Protocols targeting Alpha and Theta rhythms, in particular, have been linked to improvements in anxiety and depression (Dehghani et al., 2023; Fernández-Álvarez et al., 2022; Gadea et al., 2020; He et al., 2023; Misaki et al., 2024; Patil et al., 2023; Perez et al., 2025; Treves et al., 2025). In contrast, tVNS delivers mild electrical stimulation to the auricular branch of the vagus nerve, influencing brain regions such as the locus coeruleus and the nucleus of the solitary tract via noradrenergic pathways (Naparstek et al., 2023). Evidence suggests that tVNS can modulate neural excitability and has been explored for potential effects on anxiety and depressive symptoms, with a favorable safety profile (Austelle et al., 2025; Hilz, 2022; Kim et al., 2022; Redgrave et al., 2018; Tousif et al., 2025).

In addition to psychological outcomes, biomarkers such as salivary cortisol provide insight into neuroendocrine regulation in stress-related conditions. Dysregulated cortisol levels are often observed in anxiety and depression (Mikkelsen et al., 2021; Sitorus & Silitonga, 2025), and some studies indicate that both NF and tVNS may affect cortisol levels, although findings are inconsistent (Aliño Costa et al., 2016; Doerr et al., 2023; Hua et al., 2025; Shahyad et al., 2024).

Given their distinct mechanisms—NF engaging top–down cortical regulation and tVNS modulating bottom–up autonomic pathways—there is a theoretical rationale for combining these techniques. Such integration may enhance outcomes across psychological, physiological, and neurophysiological domains (Naparstek et al., 2023).

The present pilot study examined the individual and combined effects of NF and tVNS in healthy adults, focusing on anxiety, depressive symptom subtypes, salivary cortisol, and EEG Alpha/Theta ratio. We hypothesized that (1) NF would reduce anxiety and depressive symptoms and increase Alpha/Theta ratio, (2) tVNS would modulate neurophysiological activity and reduce psychological symptoms, and (3) the combined NF + tVNS intervention might produce synergistic effects across outcome domains.

## Methods

This pilot study was designed to generate preliminary effect sizes and identify potential mechanisms of action to inform future research and adequately powered randomized controlled trials. Given the pilot nature and small sample size (*n* = 5 per group), findings are exploratory and hypothesis-generating rather than confirmatory. The study prioritized internal validity through the rigorous standardization of procedures, while acknowledging the inherent limitations in external validity and statistical power characteristic of pilot investigations.

### Study Design

This pilot study employed a quasi-experimental design to explore the short-term effects of NF, tVNS, and their combination on psychological, neurophysiological, and biological outcomes. Twenty healthy university students were randomly assigned to one of four groups (*n* = 5 per group): NF, tVNS, combined NF + tVNS, or control. Given the small sample size, results were considered exploratory.

### Participants

Participants were 20 medication-free students (19 females, 1 male) aged 18–24 years, with no history of neurological, cardiovascular, or psychiatric conditions (including substance use disorders or seizure history). All participants provided written informed consent, and the university’s ethics committee approved the study.

The predominantly female composition of the sample (95% female, n = 19) limits the generalizability of findings to broader populations, given documented sex differences in stress response systems and HPA-axis regulation (Bangasser & Cuarenta, 2021). Baseline BAI scores ranged from 6 to 41 (M = 23.35, SD = 10.95), with most participants falling in the mild-to-moderate anxiety range rather than exhibiting clinical-level symptomatology. MCMI scores similarly reflected subclinical symptom profiles typical of university populations experiencing normative developmental stress (Sears, 1986). Future research should recruit more gender-balanced samples with clinically relevant symptom levels to better assess therapeutic potential.

### Interventions

All interventions were delivered twice weekly over four weeks. Sessions lasted 30 min (60 min for the combined NF + tVNS group) and were conducted in a quiet, controlled laboratory setting.

*NF:* The Alpha/Theta protocol (Demos, 2005) was applied using a two-channel Vilistus EEG device (England) with laptop-based software. Electrodes were placed at Cz (active), A1 (ground), and A2 (reference). Participants were guided into a relaxed eyes-closed state, and audio-visual feedback was provided to enhance Alpha (8–12 Hz) and Theta (4–7 Hz) power (Dehghani et al., 2023; Fernández-Álvarez et al., 2022; Gadea et al., 2020; He et al., 2023; Misaki et al., 2024; Patil et al., 2023; Perez et al., 2025; Treves et al., 2025). Each session began with a brief diaphragmatic breathing exercise.

*tVNS:* Stimulation was applied to the left tragus using the Parasym Device (Parasym Ltd, London, UK) with a pulse width of 200 μs, frequency of 20 Hz, and intensity adjusted to participant comfort (11–25 mA) for 30 min per session (Austelle et al., 2025; Hilz, 2022; Kim et al., 2022; Naparstek et al., 2023; Redgrave et al., 2018; Stavrakis et al., 2022; Tousif et al., 2025).

*Combined NF* + *tVNS:* Participants received NF followed immediately by tVNS within each session.

In the combined condition, NF was delivered first, followed immediately by tVNS within each session. This order was selected to allow participants to first establish cortical self-regulation through NF before receiving vagal stimulation. However, we acknowledge that this fixed sequence does not account for potential order effects or carryover between interventions. Fatigue effects or attention decrements during the second intervention may have reduced efficacy in the combined condition, and alternative sequencing (tVNS followed by NF) or counterbalanced designs should be investigated in future research.

*Control:* Participants underwent baseline and post-assessments without any active intervention.

### Outcome Measures

#### Psychological Measures

*Anxiety:* The Beck Anxiety Inventory (BAI; Beck et al., 1993) and selected scales from the Millon Clinical Multiaxial Inventory-IV (MCMI-IV) were used to assess Anxiety Syndrome (A), Post-Traumatic Stress (R), and Avoidant Personality Pattern (2A) (Mohammadi et al., 2023).

*Depression:* Three depressive subtypes from the MCMI-IV were assessed: Depressive Personality Pattern (3), Dysthymia (D), and Major Depression (Severe Clinical Syndrome, CC) (Mohammadi et al., 2023).

#### Electrophysiological Measures

*EEG:* Resting-state EEG was recorded for 5 min (eyes closed) pre- and post-intervention using the Vilistus DSU system and analyzed with Vilistus/Pro software (Version 16). Signals were sampled at 256 Hz, filtered (1–45 Hz), and analyzed using FFT to extract absolute Alpha (8–12 Hz) and Theta (4–7 Hz) power. The Alpha/Theta ratio was calculated as a marker of relaxation/attention. Real-time artifact rejection (sEMG > 5 μV, 50/60 Hz noise) was applied.

#### Biological Measures

*Morning Salivary Cortisol (MSC):* Samples were collected within 30 min of waking, stored at –20 °C, and analyzed via liquid chromatography–tandem mass spectrometry (Aliño Costa et al., 2016; Doerr et al., 2023; Hua et al., 2025; Inder et al., 2012; Shahyad et al., 2024). Participants were instructed to avoid eating, brushing teeth, or exercising prior to collection.

*Participant Instructions:* Participants received comprehensive written and verbal instructions to: (1) take nothing by mouth except water from awakening until sample collection, (2) avoid smoking, and (3) avoid physical exercise during the sampling period. Participants were informed about the objective monitoring strategy to improve sampling accuracy.

All outcome measures were assessed at two time points: baseline (pre-intervention, one week prior to the first session) and post-intervention (one week following the final session). This design enabled evaluation of overall intervention effects while minimizing participant burden in this pilot investigation. However, the absence of intermediate assessments (e.g., weekly or bi-weekly) limits our ability to characterize the time course of change or identify when treatment effects emerge.

### Missing Data

All 20 enrolled participants completed the full series of eight intervention sessions in their assigned conditions without dropout. No EEG data were excluded due to excessive artifact contamination, as real-time artifact rejection procedures maintained signal quality throughout all recording sessions.

However, not all participants completed every assessment component at both timepoints. Post-intervention data were incomplete for: salivary cortisol (*n* = 3 missing: SA039 and SA997 from Control group; 20,015 from Combined group), Beck Anxiety Inventory (*n* = 1 missing: SA039 from Control group), and MCMI-IV (*n* = 2 with incomplete data: SA974 from tVNS group missing both pre- and post-intervention assessments; SA039 from Control group missing post-intervention assessment only). Available-case analysis was employed, with exact sample sizes specified for each statistical test in the Results tables.

### Study Limitations

This pilot study did not include sham NF or sham tVNS control conditions. While the waitlist control group underwent pre- and post-intervention assessments without active intervention, the absence of sham conditions means we cannot fully exclude the contribution of expectancy effects, placebo responses, or non-specific factors (e.g., therapist attention, belief in treatment efficacy) to observed improvements in self-report psychological measures. Future adequately powered trials should incorporate sham controls (e.g., yoked NF paradigms, subthreshold tVNS intensities; Badran et al., 2018; Thibault et al., 2017) to isolate mechanism-specific effects.

### Statistical Analysis

Within-group changes from pre- to post-intervention were analyzed using the Wilcoxon Signed-Rank Test due to the small sample size and non-normal data distribution. Two-tailed *p*-values < 0.05 were considered statistically significant. Between-group comparisons were not performed due to the limited sample size. The focus was on detecting within-group trends to inform future studies. All analyses were conducted using IBM SPSS Statistics.

Exploratory correlational analyses examining associations between neurophysiological changes (ΔAlpha/Theta ratio) and psychological symptom changes were conducted separately for each intervention group using Spearman’s rank correlation coefficient (rs), which is appropriate for small samples with non-normal distributions (Bonett &Wright, 2000). For *n* = 5, the critical value for statistical significance (α = 0.05, two-tailed) is |rs|≥ 0.90; for *n* = 4, the critical value is |rs|= 1.00 (Zar, 2005).

Given the limited statistical power, these analyses are presented solely for exploratory, hypothesis-generating purposes and should be interpreted with extreme caution.

## Results

This section reports the effects of NF, tVNS, and their combination on psychological, neurophysiological, and biological outcomes. Results are organized into three subsections: within-group statistical comparisons, individual variability patterns, and exploratory correlations between neurophysiological and psychological changes.

### Descriptive Statistics and Within-Group Comparisons

Within-group changes from pre- to post-intervention were analyzed using the Wilcoxon signed-rank test due to the small sample size and non-normal data distribution. Table 1 presents descriptive statistics and statistical comparisons for all outcome measures across the four study groups (Control, NF, tVNS, and Combined).

**Table 1:** Descriptive Statistics and Within-Group Comparisons of all Measures Across all Groups

Groups	Measures	Pre-Mean	Pre- Standard Deviation	Post-Mean	Post-Standard Deviation	Cohen’s d	P-value
C	BAI	29.800	8.983	22.750	9.323	−0.72	0.068
	MSC	0.907	0.564	0.538	0.150	−0.86	0.18
	Alpha/Theta	1.842	0.285	1.710	0.248	−0.25	0.144
	MCMI_2A	73.400	32.044	85.250	8.098	+ 0.52	0.257
	MCMI_A	86.800	23.679	97.500	12.662	0.00	1.000
	MCMI_R	64.600	27.162	56.250	42.727	−0.25	0.465
	MCMI_3	77.800	15.897	87.000	9.055	+ 0.68	0.854
	MCMI_D	59.200	29.878	64.250	21.313	+ 0.19	0.102
	MCMI_CC	60.600	34.761	70.000	15.556	0.00	1.000
NF	BAI	22.000	14.765	10.000	7.616	−1.02	0.042^*^
	MSC	0.987	0.363	0.941	0.488	−0.11	0.686
	Alpha/Theta	1.532	0.139	1.670	0.227	+ 0.34	0.223
	MCMI_2A	54.800	17.456	40.600	14.258	−0.90	0.138
	MCMI_A	74.000	30.684	53.000	31.544	−0.68	0.068
	MCMI_R	44.800	33.959	33.200	31.799	−0.35	0.216
	MCMI_3	70.000	14.509	58.600	16.876	−0.73	0.066
	MCMI_D	51.600	38.004	26.600	21.675	−0.74	0.144
	MCMI_CC	53.600	39.960	20.200	32.314	−0.92	0.048^*^
tVNS	BAI	23.400	12.582	22.200	11.819	−0.10	0.686
	MSC	0.782	0.411	0.576	0.303	−0.55	0.080
	Alpha/Theta	1.630	0.191	2.046	0.322	+ 1.40	0.042^*^
	MCMI_2A	67.250	24.527	62.750	36.573	−0.14	1.000
	MCMI_A	78.750	4.787	64.000	42.903	−0.53	1.000
	MCMI_R	51.500	26.185	40.250	36.059	−0.36	0.102
	MCMI_3	67.750	25.382	78.500	19.122	+ 0.45	0.273
	MCMI_D	56.250	30.060	52.500	40.137	−0.11	0.465
	MCMI_CC	64.750	14.315	32.750	44.836	−1.06	0.144
NF + tVNS	BAI	23.000	9.618	22.400	11.502	−0.06	0.893
	MSC	0.835	0.390	0.680	0.172	−0.66	0.273
	Alpha/Theta	2.046	0.372	1.972	0.373	−0.21	0.893
	MCMI_2A	70.400	35.182	63.400	22.941	−0.23	0.500
	MCMI_A	90.800	15.353	67.400	36.094	−0.84	0.138
	MCMI_R	64.600	29.083	59.600	37.534	−0.16	0.465
	MCMI_3	84.800	14.618	81.600	15.076	−0.22	0.893
	MCMI_D	71.800	38.900	73.000	28.460	+ 0.04	0.686
	MCMI_CC	64.000	39.440	74.600	32.997	+ 0.29	0.893

All values are presented as Mean (Standard Deviation). Statistical significance was determined using the Wilcoxon signed-rank test. *p < .05. Sample sizes vary due to missing data: SA039 (Control) missing post-intervention BAI and MCMI; SA997 (Control) missing post-intervention MSC; 20,015 (Combined) missing post-intervention MSC; SA974 (tVNS) missing all MCMI assessments at both timepoints

As demonstrated in Table 1, the Control group showed no significant changes across any psychological or neurophysiological measures (all *p* > 0.05), with small-to-medium effect sizes (*|d|*= 0.00–0.86), confirming the stability of this group without any intervention. This result supports the validity of the study’s randomization process and indicates that observed changes in the intervention groups are not due to time or measurement effects alone.

**Fig. 1:**
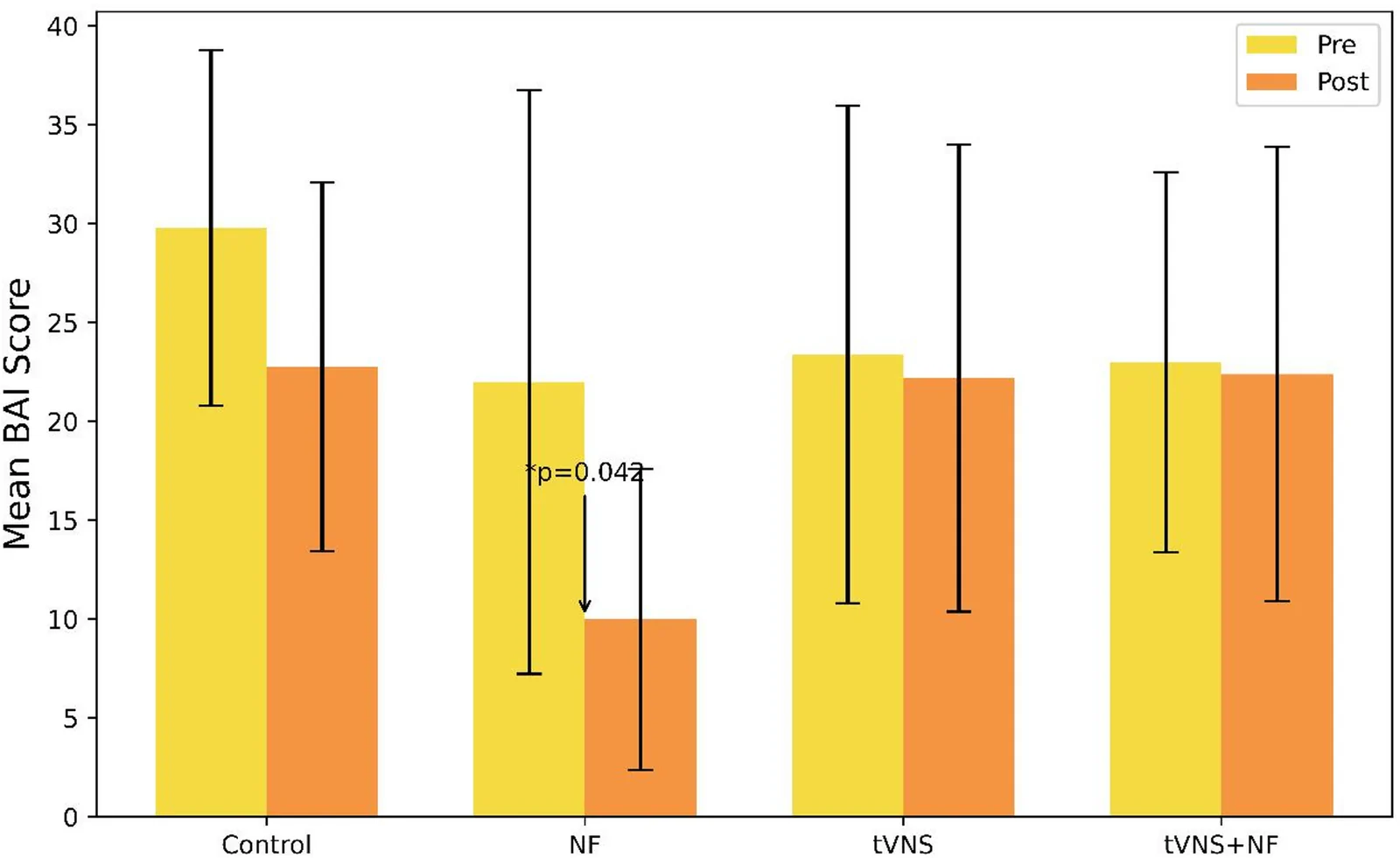
Mean changes in BAI scores across study groups from pre- to post-intervention. The NF group showed a significant reduction (*p* = 0.042) compared to tVNS, combined (NF + tVNS), and control (C) groups

**Fig. 2:**
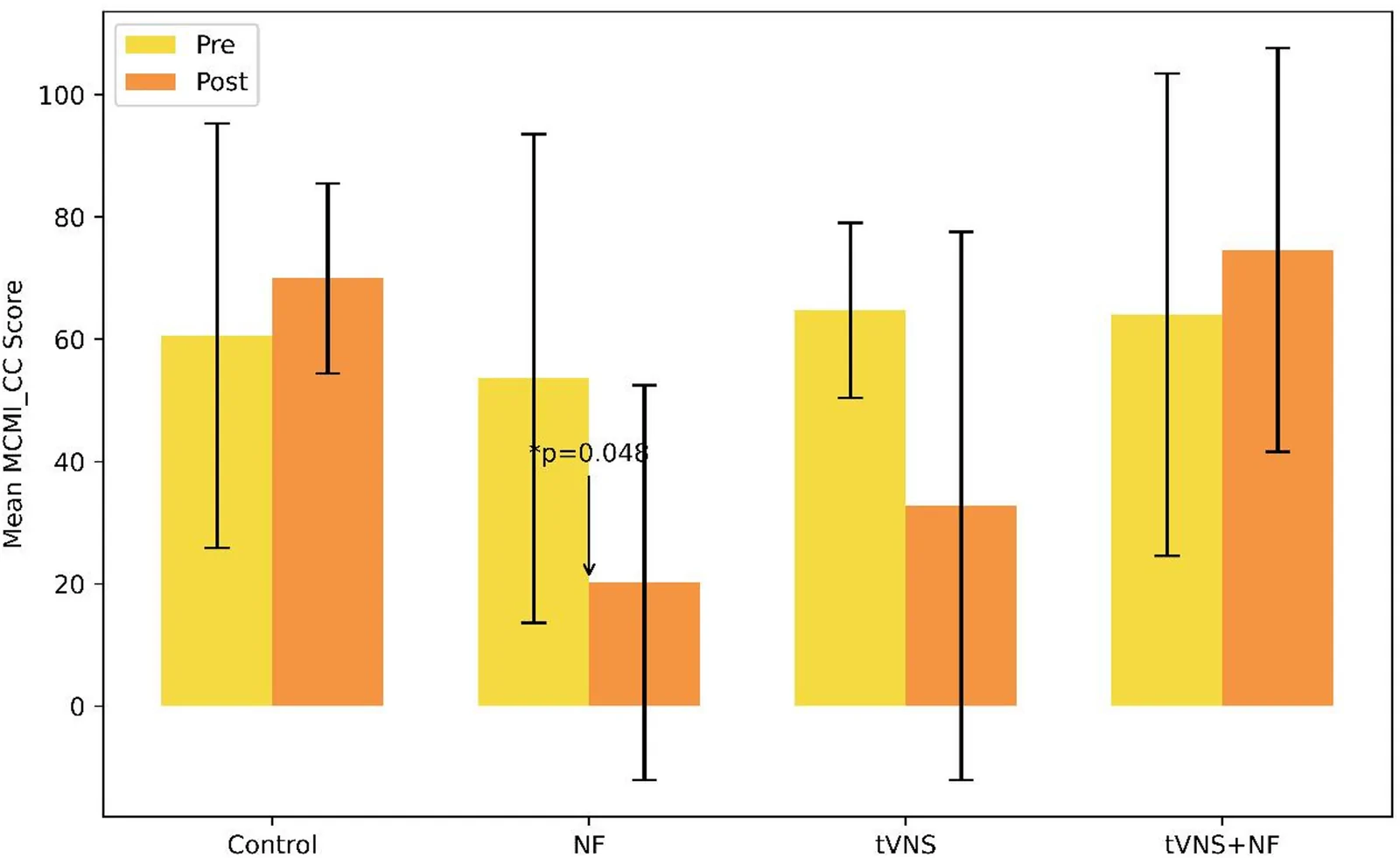
Mean changes in MCMI-CC scores across study groups from pre- to post-intervention. Significant improvement was observed in the NF group (*p* = 0.048), in contrast to increasing trends in the control and combined groups

In the NF group, the within-group analysis revealed significant improvements in specific psychological outcomes following the intervention. A statistically significant reduction was observed in anxiety levels, as measured by the BAI (*p* = 0.042, *d* = -1.02), representing a large effect size. Additionally, a significant decrease was found in the MCMI_CC subscale (*p* = 0.048, *d* = -0.92), representing a large effect size and suggesting a meaningful reduction in major depression symptomatology. While other measures, including MSC, Alpha/Theta ratio, and additional MCMI subscales, showed numerical improvements, these changes did not reach statistical significance (*p* > 0.05). Trend-level improvements were noted in MCMI_A (*p* = 0.068, *d* = -0.68) and MCMI_3 (*p* = 0.066, *d* = -0.73), representing medium-to-large effect sizes despite not reaching statistical significance. (Figs. 1 and 2).

**Fig. 3:**
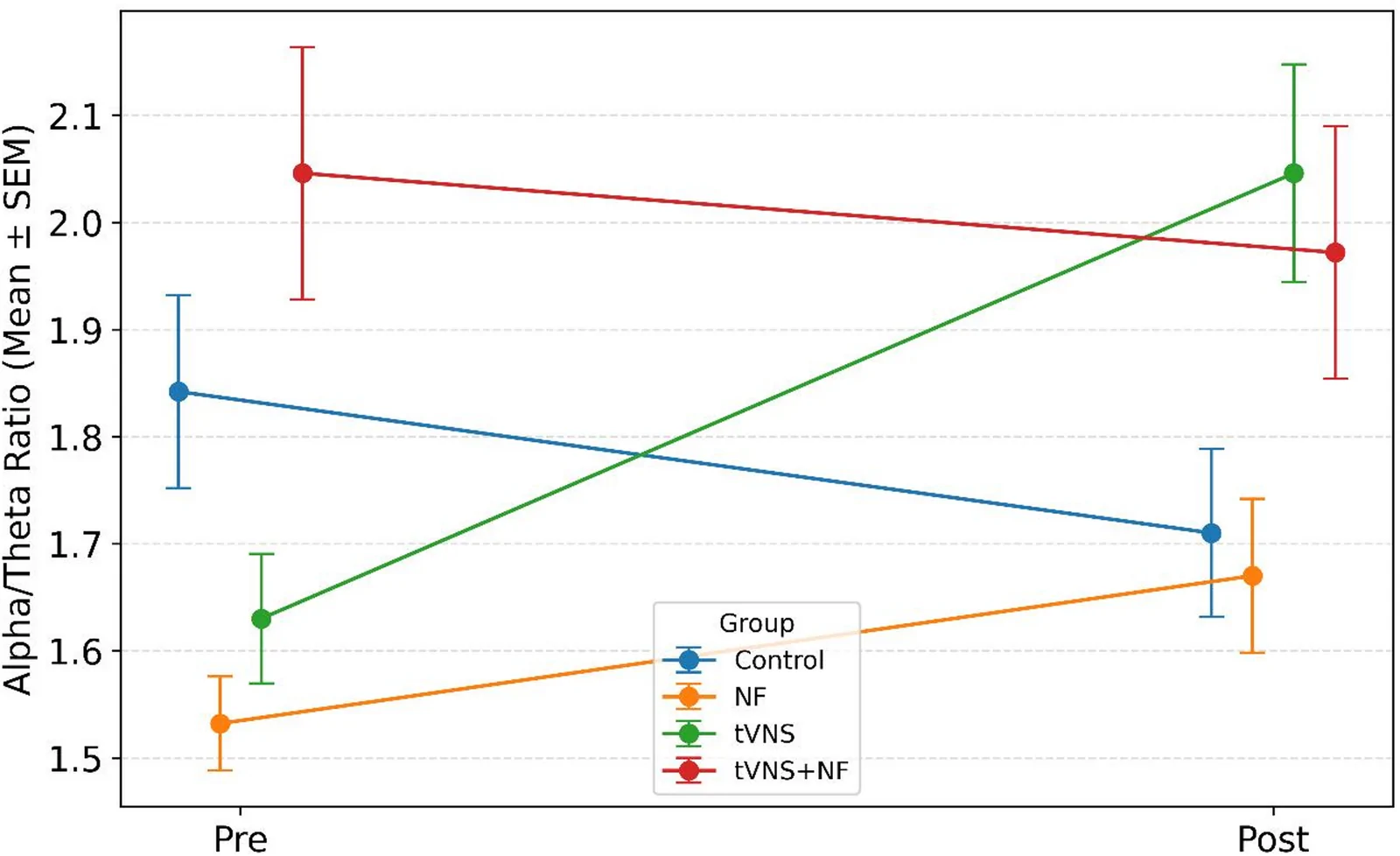
Mean changes in Alpha/Theta ratio across study groups from pre- to post-intervention. The tVNS group demonstrated a significant increase (*p* = 0.042), while the combined group showed no enhancement

In the tVNS group, results showed a statistically significant increase in the Alpha/Theta ratio (*p* = 0.042, *d* = 1.40), representing a large effect size, which may indicate a change in neurophysiological regulation. However, anxiety levels measured by BAI did not significantly change (*p* = 0.686, *d* = -0.10), with a negligible effect size. A trend-level decrease was also observed in the MSC score (*p* = 0.080, *d* = -0.55), representing a medium effect size. (Fig. 3).

**Fig. 4:**
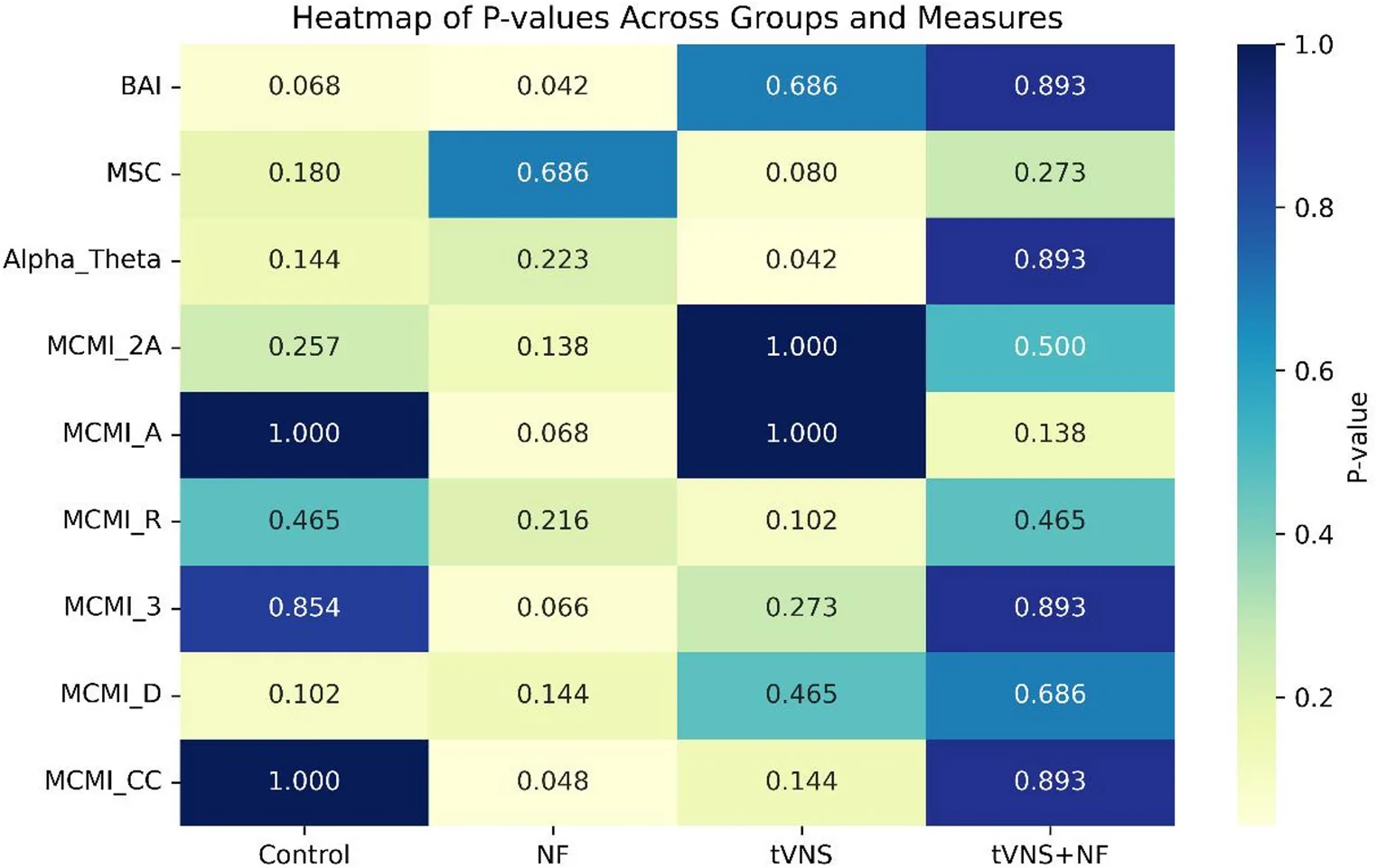
Heatmap of *p*-values across all measures, summarizing statistical significance across groups and outcome domains

Finally, the group that received the combined NF + tVNS intervention did not show any statistically significant changes in any of the measured variables. Anxiety levels, as assessed by the BAI, remained virtually unchanged (*p* = 0.893, *d* = -0.06), with a negligible effect size. Similarly, no significant differences were observed in the Alpha/Theta ratio, MSC scores, or any of the MCMI clinical subscales (*p*-values ranging from 0.138 to 0.893), with small-to-medium effect sizes (*|d|*= 0.04–0.84). These results are summarized in a heatmap of *p*-values (Fig. 4).

### Individual Variability and Response Heterogeneity

Substantial individual variability was observed across all outcome measures within each intervention group, reflecting marked heterogeneity in treatment response. In the NF group, pre-intervention BAI scores ranged from 8 to 39 (*M* = 22.00, *SD* = 14.77), indicating substantial baseline variance in anxiety severity despite homogeneous sample characteristics. Post-intervention scores ranged from 4 to 23 (*M* = 10.00, SD = 7.62), with individual change scores varying from -31points (substantial improvement) to + 4 points (slight worsening). This wide dispersion demonstrates that while group-level effects showed significant anxiety reduction (*p* = 0.042, *d* = -1.02), individual response patterns were highly variable.

Similarly, Alpha/Theta ratio changes in the tVNS group exhibited considerable heterogeneity, with individual changes ranging from + 0.03 (minimal change) to + 0.92 (large increase) despite significant group-level effects (*p* = 0.042, d = 1.40). Standard deviations for change scores were substantial relative to means across most measures (*SD*/Mean ratios typically 0.5–1.0), indicating that within-group variability approached or exceeded mean effect magnitudes. This pattern was consistent across psychological (BAI, MCMI), neurophysiological (Alpha/Theta ratio), and biological (cortisol) domains.

This pronounced individual variability likely reflects multiple factors including heterogeneous baseline characteristics, differential neural plasticity or learning capacity for NF training, varied responsiveness to vagal stimulation, and inherent measurement variability characteristic of small samples. The subclinical nature of the sample may have further contributed to variability, as individuals with minimal baseline symptoms have limited capacity for measurable improvement (floor effects), while those with moderate symptoms may respond more robustly.

### Exploratory Correlations Between Neurophysiological and Psychological Changes

Given the small within-group sample sizes (*n* = 4–5), we conducted a highly exploratory correlational analyses separately for each intervention group using Spearman’s rank correlation coefficient (rs), which is more appropriate than Pearson’s r for small samples with non-normal distributions. These analyses should be interpreted with extreme caution and are presented solely for hypothesis-generation purposes.

*Neurofeedback Group (n* = *5):* Correlations between changes in Alpha/Theta ratio (ΔAlpha/Theta) and psychological symptom changes showed varied patterns: ΔBAI (rs = 0.16, p = .800); ΔMCMI-A anxiety.

(rs = 0.46, *p* = .434); ΔMCMI-D dysthymia (rs = 0.82,* p* = .089); ΔMCMI-CC major depression (rs = 0.56, *p* = .322); ΔMCMI-3 depressive personality (rs = −0.89, *p* = .040); ΔMCMI-R PTSD (rs = 0.05, *p* = .933); ΔMCMI-2A avoidant (rs = −0.05, *p* = .935).

*tVNS Group (n* = *4–5):* Correlations showed: ΔBAI (rs = −0.50, *p* = .391, *n* = 5); ΔMCMI-A anxiety (rs = −0.20, *p* = .800, *n* = 4); ΔMCMI-D dysthymia (rs = −0.60, *p* = .400, n = 4); ΔMCMI-CC major depression (rs = 0.20, *p* = .800, *n* = 4); ΔMCMI-3 depressive personality (rs = 0.80, *p* = .200, *n* = 4); ΔMCMI-R PTSD (rs = −0.74, *p* = .262, *n* = 4); ΔMCMI-2A avoidant (rs = −0.80, *p* = .200, *n* = 4). Note: MCMI correlations are based on *n* = 4 due to participant SA974 having missing MCMI data at both timepoints.

*Combined Group (n* = *5):* Correlations showed: ΔBAI (rs = −0.70, *p* = .188); ΔMCMI-A anxiety (rs = 0.20, *p* = .747); ΔMCMI-D dysthymia (rs = 0.10, *p* = .873); ΔMCMI-CC major depression (rs = −0.50, *p* = .391);

ΔMCMI-3 depressive personality (rs = −0.60, p = .285); ΔMCMI-R PTSD (rs = −0.60, *p* = .285); ΔMCMI-2A avoidant (rs = −0.20, *p* = .747).

*Interpretation:* With *n* = 4–5 per group, these correlations lack statistical power and are subject to extreme uncertainty. For Spearman’s rs with *n* = 5, the critical value for statistical significance (α = 0.05, two-tailed) is |rs|≥ 0.90, meaning only near-perfect rank concordance would reach significance. For n = 4, only perfect.

rank concordance (|rs|= 1.00) reaches significance. None of the observed correlations reached these critical thresholds.

While one correlation in the NF group (ΔMCMI-3 depressive personality) yielded *p* = .040, the correlation coefficient (rs = −0.89) falls below the critical value of 0.90 required for *n* = 5, and should therefore be interpreted with caution as likely reflecting sampling variability rather than a reliable relationship.

The pattern of correlations did not reveal consistent relationships between neurophysiological and psychological changes within any intervention group.

Correlations ranged from strongly negative to strongly positive across groups and measures, with no discernible pattern. This absence of strong within-group correlations, combined with the dissociation of outcome domains across groups (NF: psychological improvements without significant Alpha/Theta changes; tVNS: Alpha/Theta changes without psychological improvements), suggests that the two intervention modalities may operate through distinct mechanisms rather than a unified pathway linking neurophysiological changes to psychological outcomes.

These findings should be considered strictly exploratory and hypothesis-generating. Adequately powered studies with larger samples (minimum *n* = 30–50 per group) are required to rigorously test mechanistic relationships between neurophysiological and psychological changes following neuromodulation interventions.

## Discussion

This pilot study investigated the effects of NF, tVNS, and their combination on psychological, neurophysiological, and biological outcomes in healthy adults. The findings suggest that NF and tVNS may operate through distinct mechanisms, with NF influencing psychological outcomes and tVNS modulating neurophysiological activity.

### NF: Psychological Effects

Participants in the NF group exhibited significant reductions in anxiety (BAI) and depressive symptoms (MCMI_CC), aligning with previous studies on EEG-based Alpha/Theta training (Fernández-Álvarez et al., 2022; Gadea et al., 2020; Hammond, 2005; Marzbani et al., 2016; Trambaiolli et al., 2021). Meta-analytic evidence supports large within-group effects on depressive symptoms and moderate improvements in anxiety and emotion regulation (Pindi et al., 2022; Xia et al., 2024; Barlas, 2021). Connectivity-based EEG/fMRI NF approaches may enhance emotional regulation by integrating brain-network feedback mechanisms (Dehghani et al., 2023). Even consumer-grade NF devices, such as Muse or Emotiv, produce modest yet significant reductions in self-reported stress and anxiety (Treves et al., 2025).

However, the absence of sham NF controls means we cannot definitively attribute these psychological improvements to the specific mechanism of learned Alpha/Theta self-regulation versus non-specific factors, including expectancy effects, placebo responses, therapeutic alliance, or regression to the mean. Meta-analytic evidence comparing active NF to sham controls shows smaller effect sizes than within-group comparisons (Schönenberg et al., 2017; Thibault et al., 2017), suggesting that non-specific factors contribute substantially to observed improvements. Therefore, the present findings should be interpreted as preliminary evidence warranting further investigation with a sham controls.

### tVNS: Neurophysiological Effects

The tVNS group demonstrated a significant increase in the Alpha/Theta EEG ratio, indicating enhanced relaxation or attentional regulation. These findings are consistent with prior EEG and fMRI studies demonstrating tVNS-induced modulation of cortical oscillations, including increases in delta and Alpha power, microstate dynamics, and frontal Theta activity (Briand et al., 2020; Gianlorenco et al., 2022; Jin et al., 2025; Keute et al., 2020; Konjusha et al., 2022; Ricci et al., 2020). Despite these electrophysiological changes, psychological outcomes did not significantly improve, suggesting that bottom–up modulation may require longer intervention or additional task engagement to produce observable behavioral effects.

The counterintuitive finding that tVNS but not NF increased the Alpha/Theta ratio—despite NF directly targeting this oscillatory parameter—warrants careful interpretation. Given the small sample size (*n* = 5 per group), this pattern may reflect statistical variability, as the NF group showed a non-significant trend toward an increase (*p* = 0.223, *d* = 0.34) with substantial individual variability. If the pattern is reliable, it may reflect genuinely different mechanisms: NF trains conscious, effortful self-regulation with variable performance depending on learning rate and motivation, whereas tVNS produces passive bottom-up modulation of brainstem nuclei that may induce more consistent changes in global oscillatory balance. Additionally, NF may produce state-dependent changes (enhanced Alpha/Theta only during active training) that do not generalize to resting-state recordings, whereas tVNS may induce more sustained trait-level changes in basal oscillatory patterns.

### Combined NF + tVNS: Lack of Synergy

Contrary to our hypothesis, the combined NF + tVNS intervention did not produce additive or synergistic effects. Potential explanations include suboptimal timing, sequencing, or dosing of top–down and bottom–up interventions (Ali et al., 2013; Thut et al., 2017; Zaehle et al., 2010; Bagdasaryan and Quyen, 2013; Briand et al., 2020). These findings emphasize that multimodal approaches require careful optimization to avoid interference. Beyond sequencing effects, the combined protocol may have produced counterproductive interactions between top-down and bottom-up modulation. If the nervous system is already engaged in self-directed regulation via NF, simultaneous or immediately subsequent external modulation via tVNS might disrupt this process or create competing influences on the same neural circuits. Alternatively, the extended 60-min session duration may have exceeded optimal engagement periods, with fatigue during the second intervention reducing overall effectiveness. Future studies should investigate sequential delivery or EEG-guided real-time adaptation to maximize efficacy.

### Cortisol: Null Findings

Morning salivary cortisol levels remained unchanged across all groups, likely due to the short intervention duration, healthy sample, and limited statistical power. The four-week intervention period with twice-weekly sessions (total 8 sessions) may have been insufficient to produce sustained changes in basal cortisol secretion. Studies demonstrating cortisol changes following NF or tVNS typically employ longer intervention periods (8–12 weeks) or higher session frequency (3–4 sessions per week) (Aliño Costa et al., 2016; Bretherton et al., 2019; Doerr et al., 2023; Shahyad et al., 2024). Inter-individual variability in cortisol responsiveness may have further obscured group-level effects in this small sample. Genetic polymorphisms in glucocorticoid receptor genes influence cortisol sensitivity and HPA-axis regulation (Derijk et al., 2001; Kumsta et al., 2008), creating substantial individual differences in both baseline cortisol levels and responsiveness to neuromodulatory interventions. Without pre-screening for genetic variants or cortisol reactivity phenotypes, our small sample likely included a heterogeneous mix of responders and non-responders, thereby reducing the power to detect group-level effects, even if some individuals showed meaningful changes. Additionally, the healthy, non-clinical sample with normative baseline cortisol levels may have had limited capacity for meaningful reduction (floor effects), and clinical populations with demonstrated HPA-axis dysregulation may be more likely to show intervention-related changes.

### Broader Implications and Mechanistic Insights

**Fig. 5:**
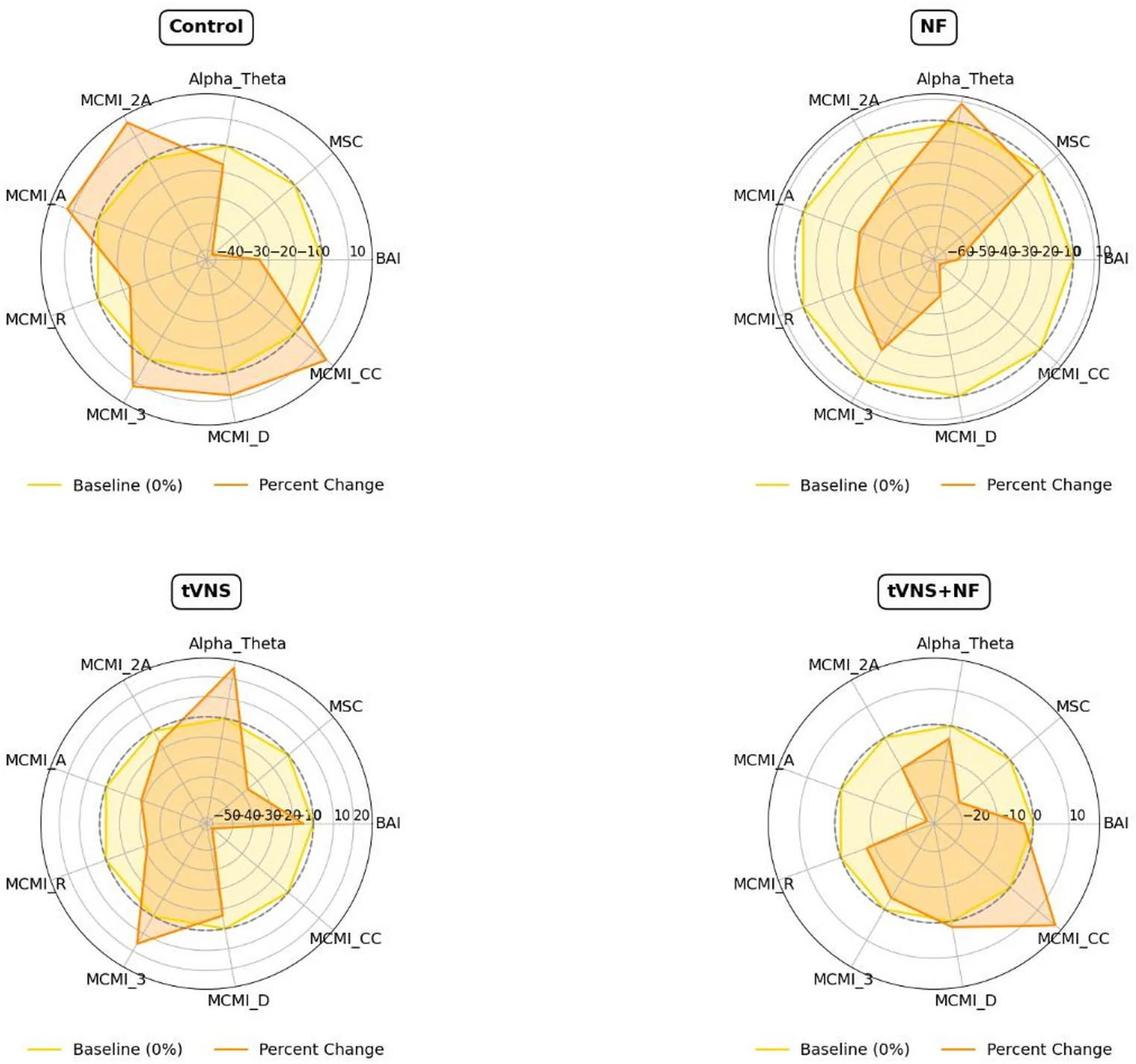
Radar plots illustrating overall intervention-specific effects across multiple outcome domains (psychological, neurophysiological, and biological)

These preliminary results suggest that NF and tVNS operate through distinct mechanisms: NF primarily affects psychological outcomes via top–down regulation, whereas tVNS modulates neurophysiological activity via bottom–up pathways. The absence of synergy in the combined group highlights the need for optimized multimodal protocols. Figure 5 illustrates intervention-specific effects across psychological, neurophysiological, and biological domains, highlighting NF’s impact on symptom reduction and tVNS’s effects on electrophysiology. This visualization emphasizes the differential pathways and supports mechanistic hypotheses for future targeted neuromodulation studies.

### Limitations

This study has several important limitations that constrain the interpretation and generalizability of findings. First and most critically, the small sample size (n = 5 per group) substantially limits statistical power and the ability to detect meaningful effects or interactions. Non-significant findings should not be interpreted as evidence of the absence of effects, but rather as insufficient evidence to detect effects that may exist. The exploratory nature of this pilot investigation means findings are hypothesis-generating rather than confirmatory and require replication in larger, adequately powered randomized controlled trials.

Second, the predominantly female composition of the sample (95%, n = 19) limits generalizability to broader populations, given documented sex differences in stress response systems, HPA-axis regulation, and prevalence of anxiety and depressive disorders. The observed effects may not generalize to male populations, individuals with diagnosed psychiatric disorders, older adults with different neuroplasticity or autonomic function, individuals with comorbid medical conditions, or diverse cultural or socioeconomic populations.

Third, the study design did not include sham NF or sham tVNS control conditions. While the waitlist control group underwent pre- and post-intervention assessments, the absence of sham conditions means observed improvements in self-report measures may partially reflect expectancy effects, placebo responses, structured attention, therapeutic alliance, or regression to the mean, in addition to or instead of specific neurophysiological mechanisms. Future confirmatory trials must incorporate appropriate sham controls to isolate mechanism-specific effects.

Fourth, outcomes were assessed only at baseline and post-intervention, with no intermediate assessments. The absence of weekly or bi-weekly measurements limits our ability to characterize the time course of change or identify when treatment effects emerge. It remains unclear whether observed improvements occur abruptly following early sessions, accumulate gradually over time, or emerge only after a minimum threshold of training. This temporal limitation applies equally to psychological, neurophysiological, and biological measures.

Fifth, in the combined condition, the fixed intervention sequence (NF always followed by tVNS) does not account for potential order effects or carryover between interventions. Fatigue effects or attention decrements during the second intervention may have reduced efficacy, Future studies should employ counterbalanced designs to isolate intervention-specific effects and determine optimal sequencing parameters for multimodal protocols.

Finally, single-channel EEG recording at Cz precluded examination of topographical distributions or regional specificity of Alpha/Theta changes, limiting mechanistic insights into whether effects are localized or distributed across cortical networks.

Despite these limitations, this pilot study provides valuable preliminary effect size estimates, demonstrates the feasibility of combined neuromodulation protocols, and identifies key methodological considerations to inform future adequately powered investigations. Future studies should incorporate larger, gender-balanced clinical samples; sham control conditions; multi-timepoint assessments; counterbalanced intervention sequencing; and multi-channel neurophysiological recordings to rigorously test the mechanistic hypotheses generated by this exploratory work.

## Conclusion

In this pilot investigation, we observed preliminary evidence suggesting that NF and tVNS may exert their effects through distinct mechanisms. While NF was associated with reductions in anxiety and depressive symptoms, consistent with top-down emotional regulation, tVNS produced a significant increase in the Alpha/Theta ratio, suggesting bottom-up modulation of neurophysiological states. The absence of synergy in the combined protocol underscores the complexity of multimodal neuromodulation and the critical importance of optimizing sequencing and intervention parameters.

However, given the small sample size (n = 5 per group), the exploratory nature of this pilot study, and the absence of sham control conditions, these findings should be interpreted cautiously as hypothesis-generating rather than definitive. The observed effects may reflect non-specific factors, including expectancy effects, placebo responses, or natural variability, in addition to specific intervention mechanisms. Replication in larger, adequately powered, sham-controlled randomized trials is essential before drawing firm conclusions about the efficacy or mechanisms of these interventions.

Despite these important caveats, this pilot work provides valuable preliminary effect size estimates (d = −1.02 for NF on anxiety; d = 1.40 for tVNS on Alpha/Theta ratio), demonstrates feasibility of combined protocols, and identifies key methodological considerations—including the need for sham controls, multi-timepoint assessments, counterbalanced sequencing, and multi-channel neurophysiological recordings—to inform the design of future rigorous investigations. These exploratory findings offer mechanistic hypotheses that, if confirmed in well-controlled studies, could contribute to the development of more targeted and effective non-invasive neuromodulation strategies in human neuroscience.

## Supplementary Information

Below is the link to the electronic supplementary material.

Supplementary Material 1

## Data Availability

The data that support the findings of this study are available from the corresponding author upon reasonable request.
